# The importance of teaching climate-health literacy in psychotherapeutic training and continuing education

**DOI:** 10.12688/f1000research.139879.2

**Published:** 2024-03-08

**Authors:** Paolo Raile

**Affiliations:** 1 Faculty of Psychotherapy Science, Sigmund Freud University, Vienna, 1020, Austria

**Keywords:** Climate-Health Literacy, Eco-Emotions, Eco-Anxiety, Climate Change, Mental Health

## Abstract

Climate-health literacy is the ability to find, access, understand, interpret, evaluate, and communicate information about the impact of climate change on human health and to make decisions and act accordingly to that information. Climate change affects people’s health in numerous ways, both directly and indirectly, *e.g.,* by increasing the risks of cardiovascular disease, infections, depression, anxiety disorders, and trauma. It is important for health professionals to understand the complex interaction between climate change and health. A teaching concept is presented that incorporates the core elements of climate-health literacy. On a first level, physical and climatological basics are taught, direct and indirect impacts of climate change on human health, climate protective measures, the psychological background of climate-protective behavior, and professional ethics. Furthermore, via self-awareness and self-reflection, the impact of climate change on the student’s mental health should be evaluated. In an advanced level, the direct and indirect impacts of climate change on mental health are taught, coping strategies, resilience, and vulnerability, as well as the role of health-care professionals in the climate crisis. In expert-level lectures, the knowledge can be deepened, and special content like activist burnout can be addressed.

## Introduction

We live in a time of crises that affect our health in many ways. One of these is the climate crisis. Climate change affects health either directly through heat waves and other extreme weather conditions or indirectly through changes in natural systems, such as the increased release of allergens or more favorable conditions for disease-transmitting organisms. The extent to which climate change affects health can be assessed in conjunction with population dynamics, as well as in economic development and health care. According to a report on climate change and health by the Austrian Panel for Climate Change ( APCC, 2018), a higher proportion of older or chronically ill people, poorer health care, and an increasing number of people with lower incomes lead to an increased societal susceptibility to climate change.

In February 2023, the research team of the “Kompetenzzentrum Klima und Gesundheit” (Competence Center for Climate and Health) wrote a research letter about health-related climate competence among health professions. The research team is part of the Gesundheit Österreich GmbH, which is the national research-and-planning institute for the health-care system, as well as the central agency for health promotion. The Republic of Austria represented by the Minister of Health is the sole shareholder of Gesundheit Österreich. In a letter about climate literacy among health-care professionals, they stated that physicians and nurses should have more literacy in dealing with climate-related health issues in their training programs ( Brugger, 2023; Brugger and Horváth, 2023).

Austrian research agencies have mentioned the psychological effects of climate change. The research letter is based on detailed reports by the APCC in 2018 and a long report and the study by Brugger in 2023. The psychological consequences are mentioned only once briefly or not at all in the report for politicians in the short forms of the APCC summary in 2018 and the research letter from Brugger and Horváth in 2023. This applies not only for Austria; the psychological consequences of climate change have a lower priority than physical ones worldwide. There are numerous studies on the effects of climate change on physical health, but few studies on its effects on mental health ( Cianconi, Betrò, and Janiri, 2020).

The purpose of this paper is to make a scientifically based plea for greater consideration of the psychological impacts related to climate change in the education and training of health professionals. For reasons of limited publication space, only one health profession will be in the focus of this paper, namely psychotherapists. They work primarily with mental-health disorders and benefit most from climate-health literacy through learning about the effects of climate change on the human psyche and *vice versa* in education and training. The impact of climate change on mental health and the impact of mental health on physical health should also be included in the training and education of other health-care professionals.

## Climate-health literacy

Climate-health literacy is a term recently used in scientific papers. It is more than a combination of climate literacy and health literacy. These two concepts are briefly defined below.

Some authors use both climate literacy and climate-change literacy interchangeably, while others distinguish between them. Dupigny-Giroux (2017) suggests that climate literacy refers to knowledge about the climate and acting accordingly to this knowledge. Climate-change literacy is more specific and refers to the knowledge and actions evolving from knowledge about the anthropogenic climate change and its impacts. This paper uses the two terms synonymously and refers to knowledge about climate change, its impacts on the planet and humans, human impacts on the environment, and complex reciprocal influences. “In other words, a climate literate individual is an expert in the principles of climate science and can assess information and communicate about climate change clearly and take responsible action towards decreasing unsustainable practices that negatively affect the environment.” ( Suhaimi and Mahmud, 2022, p. 2).

A systematic review of health-literacy literature was published in 2012. The authors created a combined model based on 17 different definitions of health literacy. This model contains three health domains and four dimensions. In short, health literacy is the ability 1) to access information on clinical issues, risk factors for health, and health-promoting factors, 2) to understand medical information and information on factors risking and promoting health, 3) to interpret and evaluate this information, and 4) to make informed decisions based on the interpreted and evaluated information ( Sørensen *et al.*, 2012).

Both the fields of health and climate are very complex, as is the concept of literacy. One reason is the difficulty of drawing a line between literacy and illiteracy. According to Suhaimi and Mahmud (2022), a climate literate is an expert on climate science. The question is, when is someone considered to be a climate-change expert, and what does this mean? The US Global Change Research Program wrote a guide on the essential principles of climate science to help individuals and communities gain climate literacy. They defined a climate-literate person as someone who “… understands the essential principles of Earth’s climate system, knows how to assess scientifically credible information about climate, communicates about climate and climate change in a meaningful way, and is able to make informed and responsible decisions with regard to actions that may affect climate.” ( US Global Change Research Program, 2009, p. 4). The difference between the former and the latter definition may simply be wording, but the question is whether a climate-literate person must understand the principles of climate science or has to be an expert in the principles of climate science. Based on the (etymologic) original meaning of the term literacy, the ability to read and write, it is obvious that a literate person does not have to be a language expert but must have knowledge of the principles of reading and writing. Therefore, the definition of the US Global Change Research Program is briefly summarized below.

Health literacy is the ability to find, access, understand, interpret, evaluate, and communicate information about health, including clinical issues and both risky and protective health factors, and to make decisions and act accordingly on that information. Climate literacy is the ability to find, access, understand, interpret, evaluate, and communicate information about climate science, including the impacts of climate change on living and non-living environments and vice versa (especially our impact on climate change), and to make decisions and act based on that information. Climate-health literacy is, therefore, the ability to find, access, understand, interpret, evaluate, and communicate information about the impact of climate change on human health and to make decisions and act accordingly on that information. The following definition of climate-health literacy based on a review of numerous studies and papers was published in 2020.

“Based on our thematic analysis, we define climate and health literacy as the degree to which an individual understands the complex relationship between climate change and human health; a climate health–literate individual can recognize direct and indirect linkages between climate change and health, communicate risks, assess data, comprehend uncertainty, and make informed and responsible personal decisions or advocate for broader policies that protect health.” ( Limaye *et al.*, 2020, p. 2185; see also Grabow *et al.*, 2023, p. 2).

The authors of this definition published seven climate-health literacy elements: 1) Root cause (what are the main causes of the anthropogenic climate crisis, which also affects our health), 2) Mechanism (how climate change affects human health), 3) Determinants (the strong bond between our environment and our health), 4) Implications (climate change worsens health disparities), 5) Interventions (How can the impact of climate change on our health be reduced?), 6) Evidence (Which evidence exists for climate-change impacts on our health and how do we get evidence?), and 7) Complexity (the enormous complexity of the connection between human health and climate change, which varies over space and time). The first two elements are so-called functional literacy, the next three elements are intermediate literacy, and the last two elements are advanced literacy ( Limaye *et al.,* 2020; Grabow *et al.,* 2023).

The focus here lies on mental health. It can be useful to create a more specific definition of mental-health literacy concerning climate. The impact of psychological issues, like stress on physical health and the impact of a physical illness on our psyche, are evident. Psychosomatics is not just a modern word to describe the impacts of the mental state on physical health and *vice versa*, but a concept of a body-mind unity ( Grassi *et al.,* 2019; Lowen, 2012). ^1^ A new concept “climate mental-health literacy” is not necessary because of the indivisible nature of physis and psyche. This paper includes mental health in the term climate-health literacy.

## The interactions between climate change and mental health

There are numerous scientific publications on the interactions between climate change and mental health. We systematically researched and reviewed articles and books on the topics of climate change and its impact on individuals and society with a special focus on the impacts on health. As a first step, we had to open up the research focus, as many articles addressed, for example, the effects of climate change on pollen and allergies, but not the effects of more severe allergies on mental health. In a second step, we researched and reviewed literature on indirect impacts of climate change on mental health like the impact of severe allergies on mental health. The focal points were formed inductively during the evaluation of the scientific articles and were adapted repeatedly during the review process. The following list was compiled from many different articles and books but does not claim to be complete.

### Direct impacts of climate change on mental health

*Extreme weather events and natural disasters (droughts, fires, floods, storms)*

Over ten years ago, the report of the Intergovernmental Panel on Climate Change (IPCC) warned of an increase in extreme weather events and natural disasters due to climate change ( IPCC, 2012). It is evident that such events can have a very negative effect on health if one is directly exposed to them. Floods and storms not only cause injuries and deaths, but also infectious diseases like cholera or food shortages, especially in countries with low adaptive capacities. Damage to the infrastructure can also hinder medical care and rescue operations ( Codjoe *et al.,* 2020; McMichael, 2015). Natural disasters can directly impact mental health. Traumatization and post-traumatic stress disorder (PTSD), anxiety disorders, depression, and other mental illnesses can be a result of directly experiencing a natural disaster or losing loved ones or possessions in a natural disaster ( Raile and Rieken, 2021). Heatwaves are also extreme weather events, but they have a different direct impact on human health.

*Increase in the number of hot days and heatwaves*

Climate change is causing a rise in average temperatures and also an increase in their duration and intensity ( Meehl and Tebaldi, 2004). Heatwaves lead to excess mortality, particularly among vulnerable groups, such as the elderly, the homeless, construction workers, pregnant women, people with pre-existing diseases, and young children ( Hajat, O’Connor, and Kosatsky, 2010; Xu *et al.,* 2016). High temperatures and heatwaves also affect mental health. There is an increased risk of suicide and hospitalization, including admissions due to mental illness, during high temperatures. Heat can also worsen psychological symptoms and can cause stress affecting both the body and the mind ( Thompson *et al.,* 2018). Health professionals can help people better cope with heatwaves by educating them about the risks and prevention measures, performing summer check-ups including medication adjustments, scheduling early morning or evening appointments, and initiating active communication with high-risk individuals ( Herrmann, 2023).

*The unpredictable future*

Almost no text addresses another direct impact of climate change on human health – the impact of its very existence as an unpredictable, lethal threat. The media reports about dystopian futures due to climate change, and billions of people worldwide are already experiencing changes in the climate and environment. The very fact that the climate crisis exists, and our future survival is uncertain creates anxiety and depression, especially if there is no hope of preventing the catastrophe ( Raile and Rieken, 2021; Raile, 2023a).

### Indirect impacts of climate change on mental health

*Air pollution and gases*

Climate change is closely connected with high CO _2_ emissions. Depending on the region, CO _2_ emissions come mainly from heating, cooking, transport, and electricity generation, especially from coal-fired power plants. The emission of CO _2_ is usually accompanied by air pollution and other factors that affect health in various ways, mainly due to respiratory and cardiovascular diseases ( Lelieveld *et al.,* 2015; Watts *et al.,* 2018). Climate change increases ozone levels, which also have a negative impact on the respiratory and cardiovascular systems ( Mücke, 2011; Watts *et al.,* 2015). Air pollution also affects mental health. There is evidence that some air pollutants, like nitric oxides, are associated with the risk of a new onset of depressive symptoms or worsening of existing depressive symptoms ( Buoli *et al.,* 2018; Yang *et al.,* 2021). Air pollution has a different influence on people with allergies.

*Pollen and allergies*

Due to climate change, the duration of the allergy season, which is starting earlier and earlier, is increasing; the amount of pollen is increasing, and new allergens are appearing. The latter is a consequence of the increase in average temperatures, which is why heat-loving plants (and their pollen) spread to higher and cooler regions ( Damialis, Traidl-Hoffmann, and Treudler, 2019; Ziello *et al.,* 2012). Higher temperatures, increased CO _2_ concentrations and air pollutants, such as ground-level ozone, also increase the allergenicity of pollen ( Beck *et al.,* 2013). The health effects of allergies on the immune system, especially on respiratory health, including symptoms like allergic rhinitis or asthma, skin diseases, and life-threatening situations like anaphylactic shock, are well known. There is also evidence for a correlation between allergies and higher depression and suicide rates, increasing anxieties, and a general reduction in quality of life ( Amritwar *et al.,* 2017; Stadler *et al.,* 2022).

*Water and food shortages*

As a result of climatic changes, especially more extreme weather events like droughts, heavy rainfall, and sea acidification, the supply of food and drinking water is hardly maintainable, especially in less privileged countries ( Kornhuber *et al.,* 2023). Besides the direct health consequences of food and water shortages (starvation, dying of thirst), long-term deficiency symptoms can also arise due to zinc, protein, and iron deficiencies ( Myers *et al.,* 2014; Zhu *et al.,* 2018). Food and water insecurity can lead to anxieties and other mental issues. Deficiencies of certain substances, such as iron, can also lead to developmental disorders which affect neurocognitive functions ( McWilliams *et al.,* 2022). General nutrient deficiencies are also correlated with severe depressive symptoms ( Owczarek *et al.,* 2022).

*Infectious diseases*

Higher temperatures accelerate the multiplication of pathogens ( Cohen *et al.,* 2020). Extreme weather events can cause water pollution ( Boudou *et al.,* 2020) increasing the risks of infectious diseases, especially in regions with lower hygienic standards for the water supply and a lack of sewage facilities ( Cissé, 2019). Insects that spread infectious diseases, like ticks and mosquitoes, reproduce faster at high temperatures and spread further. Even in Central Europe, diseases such as dengue fever, chikungunya fever, zika disease, and leishmaniasis can occur more frequently ( Thomas *et al.,* 2018; Tidman, Abela-Ridder, and de Castañeda, 2021). Such diseases also have an impact on mental health when you consider how serious illnesses make you feel. Countless papers have been published over the past three years on the effects of one of these infections, namely coronavirus disease 2019 (COVID-19), as well as countermeasures, such as social distancing, on mental health ( Panchal *et al.,* 2023; Saqib *et al.*, 2023; Witteveen *et al.,* 2023). Not only do infections have an impact on mental health; mental health status also influences healing ( Huremovic, 2019).

*Biodiversity loss*

Biodiversity is rapidly decreasing due to climate change and other human impacts, ( Raven and Wagner, 2021). Biodiversity is an essential life-support system on which our existence depends. Human health is linked to biodiversity in at least four different ways: 1) Biodiversity provides food and medicine and reduces air and water pollution. Biodiversity, especially plants, also reduces exposure to extreme heat. 2) Experiencing the natural environment reduces stress and restores our ability to concentrate. 3) Physical activities in natural environments produce greater health benefits. Biodiverse neighborhoods support social interactions and social cohesion. The natural environment also provides places to bond with others and offers transcendental experiences. 4) The natural environment can also cause harm via exposure to microorganisms causing infections, harmful wildlife, allergens, sunstroke, *etc.* ( Marselle *et al.,* 2021). The loss of natural environments can cause stress, anxieties, depressions, solastalgia (the distress due to environmental change in the home environment) ( Albrecht *et al.,* 2007) and many other mental health issues ( Raile and Rieken, 2021).

*Social factors (migration, wars, poverty)*

Many of the above-mentioned issues, the loss of the natural environment, the increase in extreme weather events, rising sea levels, and increasing water and food shortages impact society and cause an increase in poverty, migration, social unrest, and wars. In countries that are economically heavily dependent on agriculture and struggling with other socio-economic and political problems, climate change is also contributing to armed conflict ( Koubi, 2019; Smirnov *et al.,* 2022). The effects of climate migration on health can be diverse. Migrants tend to have a lower status in the host society, poorer access to the health system, and a higher risk of illness. There are many peri- and post-migration stressors which affect their mental health, like an increase in depressions, anxieties, etc. ( Byrow *et al.,* 2022; Spaas *et al.,* 2022). Climate migration can also lead to fears and divisions in the host society ( Metten and Bayerlein, 2023) as well as to aging of society in climate affected areas like coastal areas as young people migrate but older populations remain ( Hauer *et al.*, 2024). Another social impact is climate activism. Due to the insufficient climate-protection activities of most governments, concerned people become climate activists and fight for climate protection. This can also affect mental health causing, *e.g.,* activist burnout and depression ( Latkin *et al.,* 2022).

*Psychological factors (emotions, children)*

Climate change triggers a range of feelings that can be summarized as eco-emotions, like eco-anxiety, eco-worry, eco-fear, eco-despair, eco-anger, eco-guilt, eco-shame, and eco-grief. There are also positive emotions, like eco-hope ( Raile, 2023a). Eco-anger can arise, for example, when people see that politicians or corporations do not act in the interest of climate protection, but against it. Such feelings, especially when they are strong, can affect (mental) health. When the feelings affect everyday life, they can cause sufferers to seek professional help. Another impact of climate change is the rise of concerns about having children. Besides the ecological footprint that a child leaves in their lifetime, many are tormented by the gloomy prospects that they would like to spare a child ( Raile and Rieken, 2021).

### Impacts of (mental) health on climate change

*Impacts of (mental) health on climate change*

Mental health can also impact climate change. We are all called upon to do our part to protect our environment and climate. If we struggle with our mental problems, such as depression, trauma, severe anxiety, and burnout, we cannot effectively protect the climate. We can only act in the interest of the community and the environment when we are doing well ( Latkin *et al.,* 2022; Raile, 2023a). Fortunately, there are numerous activities we can do to protect the climate AND our health. One example is cycling or walking instead of driving a car. Another example is eating less meat and fast food and switching to regional products ( Herrmann, 2023). We have to take care of ourselves before we can take care of our environment and climate.

The effects of climate change on (mental) health listed here are far from complete and only give an idea of the high complexity of the interactions. A reasonably complete and sufficiently detailed review on the link between climate change and mental health would fill countless papers and books. Such literature exists (and has been cited here) and can and should be included in the education and training of health professionals, especially psychotherapists. The next section will briefly discuss psychotherapeutic education and training.

## Psychotherapeutic education in German-speaking countries with a focus on climate change

The practice of psychotherapy is not regulated by law in every country in the world. Where it is not, no law prescribes what training must be completed to work as a psychotherapist. In those countries where psychotherapy is regulated by law, there are usually training guidelines. These differ from one country to the next around the world ( Pritz, 2002). The author of this paper has mainly studied psychotherapy training in German-speaking countries. The following paragraphs particularly apply to Germany, Austria, and Switzerland. The core statement can also be adopted for other countries.

Since the new Psychotherapists Act in Germany from 2020, the path to psychotherapeutic licensure includes a polyvalent bachelor’s degree in psychology and a postgraduate master’s degree in psychotherapy, which is usually offered in combination with clinical psychology. After completing the master’s degree, one may work as a psychotherapist. Running one’s own practice is only permitted after further specialization over several years in a specific approach. The number of psychotherapeutic approaches that can be chosen is limited. Only cognitive behavioral therapy, psychodynamic psychotherapy, and systemic therapy are legally recognized in Germany. All other psychotherapeutic approaches, like gestalt therapy, can be practiced as an alternative practitioner, but not as a psychotherapist ( Bundesrepublik Deutschland, 2020; Bundespsychotherapeutenkammer, 2021).

One must complete a master’s degree in psychology in Switzerland. Following this, several years of postgraduate training as a psychotherapist can be completed in a psychotherapeutic teaching organization. In total, 42 psychotherapeutic educational institutions are accredited in Switzerland covering various psychotherapeutic approaches, such as psychoanalysis, gestalt therapy, psychodrama, person-centered psychotherapy, systemic therapy, and many more ( Schweizerische Eidgenossenschaft, 2016). Both Switzerland and Germany consider psychotherapy to be part of psychology and teach students experimental psychology right from the start. This is a view that the author has already opposed in several papers and books ( Raile, 2023a, 2023b, 2023c) and will not be discussed further here.

The Psychotherapy Act of 1990 is still valid in Austria. It prescribes a two-part training schedule to become a psychotherapist. This consists of a general propaedeutic course and a specialized course in a specific approach. In Austria, 23 psychotherapeutic approaches are currently legally recognized, including psychosynthesis, individual psychology, gestalt therapy, and cognitive behavioral therapy. Unlike in Germany or Switzerland, it is not necessary to have a degree in psychology to complete psychotherapy training; it is even possible to become a psychotherapist without a degree in Austria ( Bundesrepublik Österreich, 1990). This is expected to change in the next few years, as a new psychotherapy law is currently being prepared and is expected to be submitted for parliamentary review in the second half of 2023 ( Datler *et al.,* 2023).

When the author of this paper published the first book on eco-anxiety three years ago ( Raile and Rieken, 2021), there were no courses for psychotherapists that explicitly addressed the topic of climate change or the psychological effects of the climate crisis. There is still no dedicated course in the German-speaking world that teaches the complex interrelationships between climate change and (mental) health mentioned in the previous section in the necessary detail (Psychologists for Future, 2023, personal communication). Fortunately, there are at least individual training events:

• In February 2023, the Forum Person-Centered Psychotherapy in Austria offered a seminar on climate change and the challenges for psychotherapy ( Forum Personzentrierte Psychotherapie, 2023).
• In March 2023, the Austrian Academy for Psychology offered a more specialized seminar on the Psychology of Climate Protection ( ÖAP, 2023).
• In May 2023, the Association for the Promotion of Clinical Behaviour Therapy offered a lecture by a psychotherapist from Psychologists for Future on the climate crisis and psychotherapy ( VFKV, 2023). The same psychotherapist holds various lectures in different organizations and talks mostly about the climate crisis and psychotherapy ( *e.g.,* CIP-Akademie, 2021; Fortschritte Hamburg, 2022).
• In June 2023, the Psychotherapy Association in Germany published an information webpage on climate protection and psychotherapy. They wrote about various aspects psychotherapists can do or should consider when treating patients. Besides clinical aspects, they wrote about health prevention and political engagement ( DPtV, 2023).
• In September 2023, the German Psychology Academy offered a seminar on psychology, psychotherapy, and climate crisis. The lecture was intended to raise awareness among psychologists and psychotherapists about this important topic and to provide understanding of psychological mechanisms in connection with the climate crisis ( Deutsche Psychologen Akademie, 2023).

Organizations like Psychologists for Future offer regular seminars on climate change and psychology/psychotherapy. The Sigmund-Freud University (SFU) in Vienna also offers seminars on climate change and psychotherapy for students and licensed psychotherapists. A Climate Study Group which organizes lectures and discussions on its own initiative has been formed by psychotherapy students at the SFU ( SFU, 2023). A separate event was held in October 2022 titled “SFU Goes Climate”, where researchers from all faculties presented their contributions to researching and addressing the climate crisis ( SFU, 2022). The Sigmund-Freud University has the first concept for a two-semester course on eco-emotions, which not only teaches the basics of climate change and its complex influences on (mental) health and vice versa, but also focuses on practical psychotherapeutic work ( Raile, 2023d). Unfortunately, most German-speaking universities do not offer such training for psychotherapists, although the topic is highly relevant. The possible contents of such a training series are summarized and critically discussed in the next section.

## Teaching climate-health literacy in psychotherapeutic education – an example

When we think about creating a course for climate-health literacy, we must define our target audience. Are we talking to psychotherapists about the impact of climate change on mental health, or are we talking to other health professionals, like doctors and nurses, or are we talking to patients, or to the general public? Depending on the target group, the form of communication (use of technical vocabulary) or the content (assumption of prior knowledge) must be adapted. While the general population benefits more from learning about the mechanisms and effects of the climate crisis and individual coping strategies, psychotherapists benefit more from learning about the climate-specific aspects of mental health and how to adequately deal with those in practice. The following paragraphs present a concept of what such a training course could look like. The concept was developed by the author taking into account the theoretical foundations outlined in the previous section and in exchange with students ( *e.g.*, the Climate Study Group of Sigmund Freud University), psychotherapy colleagues ( *e.g.*, the co-author of the monograph on Eco-Anxiety) and experts ( *e.g.*, from Psychologists For Future). A modular and sequential three-level course is being considered with seminars for beginners, advanced professionals, and experts. The explanations below include the contents of the individual courses. The didactic, the way of conveying the contents, is not explicitly described, and can be freely chosen by the lecturers, whereby a mixture of frontal presentations and interactive elements is recommended.

### Beginners

*Physical and climatologic basics*

First, it is important to make the physical and climatologic basics of climate change as understandable as possible to the participants. Not all people are aware of the basic mechanisms that lead to global warming, the increase in extreme weather events, and other impacts ( Dupigny-Giroux, 2017; US Global Change Research Program, 2009). Since psychotherapists do not need any special expertise here, this part can be taught in a condensed form.

*Direct and indirect impacts of climate change on human health*

This could be an overview seminar on the impacts of climate change on human health as explained in the chapter *impact of climate change on the psyche and vice versa.* The seminar should provide a broad but not necessarily profound knowledge about the connections between climate change and human health. Part of the lecture should contain explicit information about the impact of climate change on mental health since the target group consists of psychotherapists ( Limaye *et al.*, 2020; Grabow *et al.*, 2023). Case studies from practice are useful.

*Self-awareness and self-reflection*

For psychotherapists, self-awareness and self-reflection are an essential part of their training, practice, and professional identity. The professional reflection of one’s own techniques and interventions in therapy, one’s own strengths and weaknesses and factors influencing the treatment accompanies them in their everyday professional life ( Strauß and Taeger, 2021). It is particularly important for psychotherapists to reflect on their own role in the face of the climate crisis and to critically consider their actions and possibilities for action both within and outside the treatment setting. Such a seminar should be organized as a self-awareness group.

*Climate protection, psychological background of protective behavior, and professional ethics*

This seminar is about climate protection and the psychological factors influencing protective behavior. Psychotherapists should not only know what everyone can do to protect the climate but should also have knowledge of important psychological inhibitors and enhancers of being actively protective, like established habits or fears. They also should think about and discuss their responsibilities, what they can do, what they should do, and what they must do to protect the climate and what are they not allowed to do because of their professional roles as psychotherapists ( Raile, 2023a).

### Advanced

*The direct and indirect impact of climate change on (mental) health*

While the beginners’ seminar on the same topic focuses on an overview of the many interactions between climate change and (mental) health, individual factors are dealt with in more detail in this course. The aim is to provide more in-depth knowledge ( *e.g.*, Codjoe *et al.,* 2020; Kornhuber *et al.*, 2023; Watts *et al.*, 2018). Either a multi-part seminar or a weekend seminar could be offered and arranged so that individual influencing factors can be addressed in detail.

Eco-emotions: In the advanced seminar on eco-emotions, the wide range of emotions related to the climate crisis is extensively covered. Psychotherapists should not only gain knowledge about the triggers, but also learn about the characteristics that distinguish eco-emotions from other feelings ( Raile, 2023a, 2023d). Depending on the scope of the seminar, individual eco-emotions like eco-anxiety can be discussed in more detail and case studies can be presented.

*Coping and resilience in the climate crisis*

People who suffer from such strong eco-emotions (like eco-anxiety) that affect their everyday lives, do not always come to psychotherapeutic practice. They find other coping strategies. These can be used by psychotherapists to offer their patients more comprehensive ways of dealing with their feelings ( Raile and Rieken, 2021). The content can be connected meaningfully with the termini climate vulnerability and climate resilience, the reason why some people suffer more from the psychological effects of the climate crisis than others. Furthermore, psychotherapists should be aware of climate precarity, which means that inequalities in socio-economic, geographical, health, and demographic factors, as well as social and economic resilience have an impact on the effects of climate change on people. Consequently, this may also result in higher anxiety. Being aware of these dynamics and factors that impact the risk of a patient is quite important for psychotherapists as it gives them an understanding of what causes their eco-emotions and perhaps ways how these particular people can engage in protective behavior.

*The role of psychotherapists in the climate crisis*

Based on the seminars on self-awareness and ethics, psychotherapists should also become aware of their own role in the climate crisis ( Orange, 2016). First, as role models regarding climate protection; second, as persons who can educate people about climate change and how to overcome factors inhibiting individual climate- protection measures; third, as mental-health experts who can implement preventive as well as curative measures.

### Experts

The seminars in the experts’ section can be very different and adapted according to the target group. Different psychotherapeutic approaches and their treatment strategies for *e.g.,* severe cases of eco-anxiety, eco-traumas, and activist burn out can be addressed as well as the psychosocial care system and regional support centers for those affected by direct or indirect impacts of the climate crisis. Content on the cooperation of different professional groups in the sense of improved prevention or education is also conceivable, as well as further expertise in climate protection and ethics.

## Discussion

The concept presented here does not contain all the points dealt with in the previous section because not all of them are equally relevant for psychotherapists. A separate seminar for the impact of climate change induced infections on mental health may be more relevant for medical doctors but less relevant for psychotherapists due to their primary focus on mental health. However, it is important that mental-health professionals know about these connections and can communicate them. This makes the concept vulnerable to critical review, as other experts may see other contents as more important. Various seminar providers may also select contents according to specific interests to attract as many paying participants as possible. Other education and training providers may delete contents due to a limited budget or (in their view) a lack of relevance for psychotherapists. Regardless of the reasons for the deletions or the choice of contents, it is an important step forward if such topics are taught at all in psychotherapeutic education and training. In this regard, even a small step in this direction is a success in the fight against the advancing climate crisis and for people’s mental health in times of climate crisis.

Especially the teaching of contents like eco-emotions can be criticized by psychotherapists since dealing with emotions is part of psychotherapeutic training, anyway. This criticism can be countered in three ways: First, contents like crisis intervention or trauma therapy are also part of psychotherapeutic training, but not always sufficient to work in depth with severely traumatized people. Further training and specialization are never pointless, but relevant, for example, to get to know the specific characteristics of eco-emotions in depth. Second, pathological emotions, such as generalized anxiety disorder, are different from eco-anxiety, which can also display similar symptoms. The difference is that the threat, *i.e.,* the climate crisis, is real, and the feelings of anxiety are basically adequate. Therefore, in addition to working on the feelings, we also must work on dealing with the causes. Contextual knowledge is of great importance here, which is why further training is important. Third, such training serves to sensitize psychotherapists to the climate crisis and its effects on the human psyche. This is not only important for psychotherapeutic work, but also for their own mental health because psychotherapists can also experience eco-emotions, such as fear or anger ( Raile, 2023d).

## Conclusions and a plea for teaching climate-health literacy to health-care professionals

Climate change is steadily progressing, and humanity does not presently seem able to stop it effectively. The consequences are severe and affect not only our planet and ecosystems, but also our health. For the sake of our health and because we can only effectively protect the climate if we protect our health, it is important that health-care professionals are informed about the effects of climate change. It is important that health-care professionals, presented here using the example of psychotherapists, have climate-health literacy, *i.e.,* knowledge of the complex mechanisms and interactions between climate change and health.

As most examples for psychotherapy education in this paper are from German-speaking countries, individuals and organizations offering psychotherapy training in other parts of the world may adapt the concept due to very different educational systems or political context. Since climate change is a global threat which affects people’s mental health, all psychotherapists and all institutions offering training and continuing education for psychotherapists and all other health-care professions are recommended to include these contents with any necessary adjustments in their curricula, to offer seminars, and to provide further information on these topics.

## Data Availability

No data are associated with this article.

## Footnotes

^1^
The question of body-mind duality *vs.* body-mind unity is a millennia-old philosophical question that has still not been clarified. The author of this paper adheres to the body-mind unity definition.
