# Supplementary material for: The importance of teaching climate-health literacy in psychotherapeutic training and continuing education
Source: F1000Res. 2024 Mar 8;12:982. Originally published 2023 Aug 15. [Version 2] doi: 10.12688/f1000research.139879.2 (PMC11019290; doi:10.12688/f1000research.139879.2)
Supplement: Peer review history

# Reviewer report: Reviewer response for version 2

**DOI:** 10.5256/f1000research.163365.r254078

**Published:** 2024-04-15

**Authors:** Melanie Boeckmann, Lira Ramadani

**Affiliations:** 1 Department of Global Health, University of Bremen, Bremen, Germany; 2 Universitat Bielefeld, Bielefeld, North Rhine-Westphalia, Germany

I can confirm and say that all is okay now for this article.

Is the topic of the opinion article discussed accurately in the context of the current literature?

Yes

Are arguments sufficiently supported by evidence from the published literature?

Partly

Are all factual statements correct and adequately supported by citations?

Yes

Are the conclusions drawn balanced and justified on the basis of the presented arguments?

Yes

Reviewer Expertise:

Climate change education, climate change and health

We confirm that we have read this submission and believe that we have an appropriate level of expertise to confirm that it is of an acceptable scientific standard.

# Reviewer report: Reviewer response for version 2

**DOI:** 10.5256/f1000research.163365.r254079

**Published:** 2024-03-13

**Authors:** James Irlam

**Affiliations:** 1 School of Public Health, University of Cape Town, Rondebosch, Western Cape, South Africa

My comments and edits have been addressed by the authors thanks.

 I do query though why there are 8 instances of (mental), as I don't see the need for the parentheses ().

 Thanks

 James Irlam

Is the topic of the opinion article discussed accurately in the context of the current literature?

Partly

Are arguments sufficiently supported by evidence from the published literature?

Partly

Are all factual statements correct and adequately supported by citations?

Partly

Are the conclusions drawn balanced and justified on the basis of the presented arguments?

Partly

Reviewer Expertise:

Education for Sustainable Healthcare

I confirm that I have read this submission and believe that I have an appropriate level of expertise to confirm that it is of an acceptable scientific standard.

# Reviewer report: Reviewer response for version 1

**DOI:** 10.5256/f1000research.153192.r229388

**Published:** 2024-02-26

**Authors:** James Irlam

**Affiliations:** 1 School of Public Health, University of Cape Town, Rondebosch, Western Cape, South Africa

The article is generally well written, but it lacks even a brief description of the search strategy, and requires a discussion of its potentially limited applicability to psycho-education outside of German-speaking countries, and the implications for further research.

 Please see my comments plus the language and grammar corrections in the attached PDF linked here.

Is the topic of the opinion article discussed accurately in the context of the current literature?

Partly

Are arguments sufficiently supported by evidence from the published literature?

Partly

Are all factual statements correct and adequately supported by citations?

Partly

Are the conclusions drawn balanced and justified on the basis of the presented arguments?

Partly

Reviewer Expertise:

Education for Sustainable Healthcare

I confirm that I have read this submission and believe that I have an appropriate level of expertise to confirm that it is of an acceptable scientific standard, however I have significant reservations, as outlined above.

# Response

**Published:** 2024-03-03

**Authors:** Paolo Raile

**Affiliations:** Faculty of Psychotherapy Scien, Sigmund Freud University, Vienna, Austria

Thank you very much for your valuable time, your comments, and your corrections. I implemented nearly all of them into the new version of the manuscript (but not "the" in Fridays for Future as you recommended) and am very thankful for the high improvements due to your suggestions. I enhanced the abstract, added the limitation in the last paragraph, and added a short paragraph on how I conducted the research/reviews. Thank you once again for your time!

# Reviewer report: Reviewer response for version 1

**DOI:** 10.5256/f1000research.153192.r212179

**Published:** 2023-10-17

**Authors:** Melanie Boeckmann, Lira Ramadani

**Affiliations:** 1 Department of Global Health, University of Bremen, Bremen, Germany; 2 Universitat Bielefeld, Bielefeld, North Rhine-Westphalia, Germany

Content-wise suggestions:

 For sake of completeness, the line “Heatwaves lead to excess mortality, particularly among vulnerable groups, such as the elderly, the homeless, construction workers, pregnant women, and young children”, should also include people with pre-existing diseases.

 Some concepts could enhance the arguments made in certain sections. For example,  in the “Social factors (migration, wars, poverty)” paragraph, including the concept of “climate migration” could enhance the arguments made.

 Similarly, in the “Coping and resilience in the climate crisis”, it would be also important for climate vulnerability/ climate resilience to be added to the curricula. Psychotherapists should be aware that certain people due to socioeconomic, geographic, heath and demographic factors are more impacted by climate change than others. Consequently, this may also result in higher anxiety.

 The concept of “climate precarity” is rooted in the recognition of this inequality, which holds that class, social, labour and/or gender inequities amplify negative impacts from climate change. This concept could also enhance this paragraph. Additionally, from a community or systems level, namely the instrastructure and ecosystem resilience; social/economic resilience and climate adaptation measures also influence the level of resilience. Being aware of these dynamics and factors that impact the risk of a patient is quite important for psychotherapists as it gives them an understanding of what causes their eco-emotions and perhaps ways how these particular people can engage in protective behavior.

 Additionally, some minor grammatical errors are noted below:

 Line to “…health either directly in heat…”

 Suggestion: “…directly through heat…”

 Sentence: “People who suffer from such strong eco-emotions (like eco-anxiety) that *they affect* everyday life do not always come to psychotherapeutic practice.”

 Suggestion: “…that affect their everyday lives, do not…”

Is the topic of the opinion article discussed accurately in the context of the current literature?

Yes

Are arguments sufficiently supported by evidence from the published literature?

Partly

Are all factual statements correct and adequately supported by citations?

Yes

Are the conclusions drawn balanced and justified on the basis of the presented arguments?

Yes

Reviewer Expertise:

Climate change education, climate change and health

We confirm that we have read this submission and believe that we have an appropriate level of expertise to confirm that it is of an acceptable scientific standard, however we have significant reservations, as outlined above.

# Response

**Published:** 2024-03-03

**Authors:** Paolo Raile

**Affiliations:** Faculty of Psychotherapy Scien, Sigmund Freud University, Vienna, Austria

Thank you very much for your valuable time, your comments, and your corrections. I implemented all of them into the new version of the manuscript and am very thankful for the improvements due to your suggestions.
